# Power modulation of electroencephalogram mu and beta frequency depends on perceived level of observed actions

**DOI:** 10.1002/brb3.494

**Published:** 2016-05-24

**Authors:** Shiri Simon, Roy Mukamel

**Affiliations:** ^1^ Sagol School of NeuroscienceTel‐Aviv UniversityTel Aviv6997801Israel; ^2^ School of Psychological SciencesTel‐Aviv UniversityTel Aviv6997801Israel

**Keywords:** Action observation, conscious perception, electroencephalogram, mirror neuron system

## Abstract

**Introduction:**

The ability to understand actions and intentions of others is of great importance to social relationships and is associated with the mirror neuron system of the human brain. Whether conscious perception of specific actions is necessary to trigger activity in this system, or alternatively whether this response is independent of conscious perception is not known.

**Methods:**

We addressed this issue by rendering videos of right hand movements invisible to conscious perception, and measuring electroencephalogram (EEG) power suppression in the mu (8–13 Hz) and beta (15–25 Hz) range as index corresponding to the magnitude of mirror neuron activity.

**Results:**

In the beta range over bilateral sensorimotor sites, we find that suppression indices follow the reported perceptual level of subjects with stronger suppression for consciously perceived trials. Furthermore, in the nonperceived trials, oscillation power is significantly suppressed relative to baseline. In the low mu range (8–10 Hz), oscillation power over the left sensorimotor site is significantly more suppressed in the consciously perceived versus nonperceived trials.

**Conclusions:**

Our data suggest that the intensity of mirror system responses during action observation decreases with the observers' perception level yet remains significant during observation of invisible actions. Such subliminal activity could help explain phenomena such as covert imitation.

## Introduction

As social beings we constantly interact and communicate while relying on our ability to understand the actions and intentions of others. This ability is of great importance for survival and is associated with the Mirror Neuron System of the brain. Mirror neurons are a particular class of visuo‐motor neurons that discharge not only when executing an action but also when passively observing a similar action being executed by someone else (monkey or human) (Cattaneo et al. 2009; Rizzolatti and Sinigaglia 2010). These neurons were originally discovered using single‐cell recordings in sector F5 of the ventral premotor cortex of macaque monkeys (Dipellegrino et al. 1992; Gallese et al. 1996; Rizzolatti et al. 1996). Since the original discovery, the existence of mirror neurons in other regions of the human and nonhuman motor pathway (including parietal, and primary motor cortex) has been demonstrated (Filimon et al. 2007; Tkach et al. 2007; Chong et al. 2008; Dushanova and Donoghue 2010; Mukamel et al. 2010; Kilner and Lemon 2013; Vigneswaran et al. 2013).

Physiological studies in primates have shown that similar sensory input (e.g., a hand reaching behind a screen obscuring its end point) evokes differential mirror neuron activity depending on context (e.g., whether there is an object behind the screen or not) (Umilta et al. 2001). Additionally, during observation of similar actions, mirror neurons in inferior parietal lobe (IPL), inferior frontal gyrus (IFG), and ventral premotor cortex, have been shown to respond differentially depending solely on the goal of the action (Fogassi et al. 2005; Iacoboni et al. 2005). The idea that fronto‐parietal mirror neurons code the goal of an action rather than the action's low‐level kinematics is further supported by a study of Umilta and colleges, showing that different actions having the same goal evoke similar neural response (Umilta et al. 2008). Mirror neurons have also been shown to respond not only to visual input but also to auditory cues associated with motor acts [e.g., the sound of breaking a peanut (Kohler et al. 2002; Keysers et al. 2003)]. Taken together, it has been suggested that the activity of mirror neurons holds information regarding action goals even in the absence of complete sensory information describing it. Therefor it seems that the activity of mirror neurons in fronto‐parietal circuits is loosely tied to the physical attributes of sensory input and more sensitive to the goals of perceived actions. Although understanding of goals requires conscious perception of actions, it is not yet known to what extent activity in the mirror system depends on the degree of conscious perception of an action. Behavioral studies in humans indeed suggest that an unconscious process of imitation takes place during observation of actions performed by others, resulting in an increased tendency to perform similar actions (e.g., social contagion/the chameleon effect, Chartrand and Bargh 1999). Such implicit imitation could be the result of mirror neuron activity (Hogeveen and Obhi 2012; Cross and Iacoboni 2014).

At the physiological level, suppression of oscillatory activity within the mu (8–13 Hz) and beta (15–25 Hz) frequency bands over sensory motor regions has been associated with action execution. Similar suppression has been reported also during action observation and thus taken as an index to infer mirror neuron activity (Cochin et al. 1998; Hari et al. 1998; Perry and Bentin 2009; Arnstein et al. 2011; Fren‐kel‐Toledo et al. 2013; for review see Pineda 2005). In agreement with previous literature, we used these indices to probe the activity of the mirror neuron system and examine the level of mu and beta suppression with respect to the level of conscious action perception.

## Methods

### Participants

Nineteen healthy, right‐handed adults (7 males) ranging from the ages of 19–33 participated in this study (Mean = 24.58, SD = ±2.98). All the subjects were right handed, and had normal or corrected to normal vision. Participants were recruited from the general population of students at Tel Aviv University and were compensated for their participation with either course credit or payment. Prior to the experiment, all participants provided written informed consent to participate. The study was approved, and conformed to the guidelines set by the Tel Aviv University Ethical committee.

### Stimuli

**Figure 1:**
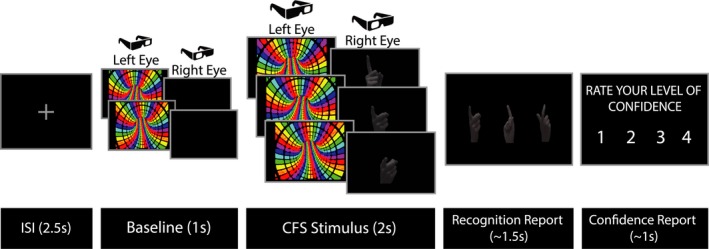
Experimental Design. During each trial two separate video clips were presented in an interleaved fashion such that odd frames (corresponding with clip 1) were presented to one eye, and even frames (corresponding with clip 2) were presented to the other eye. Clip 1 consisted of 3 sec masking video, whereas clip 2 consisted of a black screen for 1 sec followed by 2 sec of a target video. The first second of each trial was used as baseline for the analysis. At the end of each trial, participants reported which action was presented and their confidence level on a scale from 1 to 4.

We modified the Continuous Flash Suppression (CFS) paradigm (Tsuchiya and Koch 2005) to render action videos (rather than static images) invisible to conscious perception (For experimental design, see Fig. 1). The CFS procedure allows masking from visual awareness an otherwise visible stimulus presented to one eye, by simultaneously presenting strong dynamic noise to the opposite eye. Target and masking stimuli were presented at the center of a 3D monitor (Samsung led s23a950d, 120 Hz refresh rate) at 17 cm^2^ size and approximately 70 cm from the participants' eyes. Active shutter glasses that were synchronized to the monitor enabled exclusive presentation of odd frames only to one eye and even frames to the other eye. Since target and mask video frames were presented in alternate sequence mode (i.e., one frame from each video in turn), odd frames (corresponding to the mask stimulus) were presented to one eye and the even frames (corresponding to the target stimulus) were presented to the other eye. The CFS display consisted of one of three grayscale target videos of different hand movements and one of three different masking videos of colored Mondrian patterns. During each trial one eye was presented with 3 sec masking video (180 mask frames) and the other eye was presented with a black screen for 1 sec (60 frames) followed by 2 sec of the target video (another 120 target frames). The first second of each CFS display in each trial was used as baseline for the analysis.

### Task

Each trial started with the presentation of a fixation point (“+”) that lasted 2.5 sec followed by the 3 sec of CFS display. At the end of each trial participants reported their level of conscious perception. First, they were asked to report which of the three actions was presented, or guess in case they did not consciously perceived it. The participants reported their perception level by two means. First, by pressing one of three buttons corresponding to representative frames taken from the target action video presented on the screen. The mapping between buttons and action video frames was randomized across trials in order to avoid motor preparation which has been shown to result in mu and beta suppression (Pfurtscheller et al. 1996; Ohara et al. 2000; Rektor et al. 2006). Second, the participants reported their confidence level, namely to what extent they are confident in their report, on a scale from 1 to 4. Participants were asked to report “4” in case they could perceived a sequence of dynamic movement and were sure which action out of the three was displayed. A report of “1” in confidence level corresponded to cases in which they did not perceive the movement at all and were forced to guess which action was displayed. In case they could perceive the action based on a single frame or a flash of an image they were asked to report an intermediate level (“2” or “3”) of confidence.

### Procedure

The experiment included four blocks, each consisted of 75 trials. For each participant we first ran a behavioral pretest to set the level of perception to be as close to 50%. In the behavioral pretest, we presented each of the three hand movement trials five times at six different levels of brightness (ranging from 35 to 220 in grayscale). The optimal brightness chosen for the main experiment was the brightness that generated the most balanced distribution across confidence levels with at least three trials with confidence level 1 and confidence level 4 respectively. Subjects who did not meet these criteria in any brightness level were excluded from participating in the experiment.

### Data acquisition

We used a Biosemi Active Two EEG recording system (Biosemi B. V., Amsterdam, the Netherlands). Data were recorded from 64 scalp‐electrodes at locations of the extended 10–20 system, as well as from two electrodes placed on the left and right mastoids. The horizontal electro‐oculogram (EOG) was recorded from electrodes placed 1 cm to the left and right of the eye to detect horizontal eye movement, and the vertical EOG was recorded from an electrode beneath the left eye to detect blinks and vertical eye movements. The single‐ended voltage was recorded between each electrode site and a common mode sense electrode (CMS/DRL). Data were sampled and digitized at 256 Hz.

### Data analysis

We focused on the modulations of mu (8–13 Hz) and beta (15–25 Hz) rhythms measured over the sensorimotor cortex. Particularly, mu suppression in the range 8–10 Hz was found to be more suppressed during action observation than action execution (Frenkel‐Toledo et al. 2013). Therefore, mu suppression indices were computed separately for the lower mu frequency range (8–10 Hz) and the higher mu frequency range (11–13 Hz).

#### Preprocessing

Offline signal processing and analysis was performed using EEGLAB Toolbox: RRID:SCR_007292 (Delorme and Makeig, 2004) version 13.0.1 and custom MATLAB scripts: RRID:SCR_001622. All EEG signals were referenced offline to the average of the left and right mastoids and bandpass filtered between 0.5 and 40 Hz. The continuous data were segmented into epochs from −1000 to +2000 ms relative to onset of the target stimuli. EEG deflections resulting from eye movements and blinks were corrected using an ICA procedure (Jung et al. 2000). Epochs with artifacts exceeding ±100 *μ*V amplitude in the relevant electrodes were rejected. Trials were classified to conditions based on the participants' accuracy and their reports of confidence level.

#### Event‐related spectral perturbations

The integrated power in the 8–13 Hz and 15–25 Hz frequency ranges was computed using wavelet analysis. For each trial in each subject, we computed the logarithm of the power (from 500 to 2000 ms post target stimulus) relative to power during baseline (from 500 to 0 ms pre target stimulus). The suppression indices for each condition were then calculated by averaging across subjects the single‐trial log ratios values from equal number of trials for each condition. A negative log ratio indicates a suppression in EEG oscillations amplitude relative to baseline, whereas positive log ratio indicates enhancement. Suppression indices were computed at two central sites, C3 and C4, which best reflect changes in mu and beta rhythms (Pineda 2005). As control, these measures were also computed at two occipital sites, O1 and O2, where alpha (8–13 Hz) rhythms are strongest. Suppression of occipital alpha is induced during perceptual events (Pfurtscheller et al. 1994; Krause et al. 1996) and increased demands of attention (Klimesch 1996; Thut et al. 2006; Palva and Palva 2007; Sauseng and Klimesch 2008). Since the perception of our target may capture participants' attention more than the Mondrian patterns (in the case of nonperceived trials), both occipital alpha and sensorimotor mu are reported.

## Results

Two subjects had too few nonperceived trials (<25 trials) and were therefore excluded from further analysis.

### Behavior

**Figure 2:**
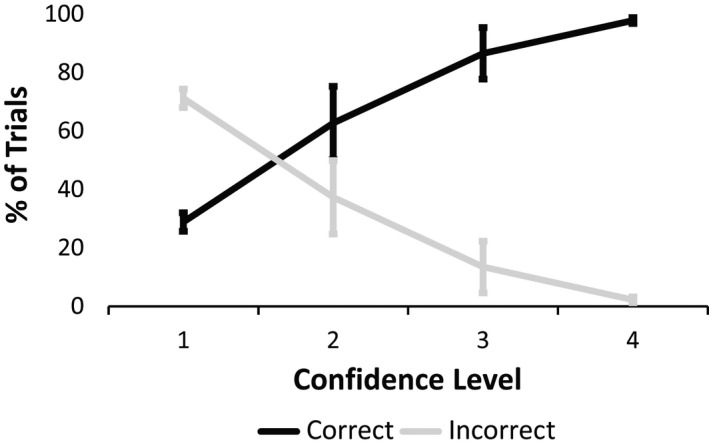
Behavioral Results. Correct reports corresponded to higher confidence levels, whereas incorrect reports corresponded to lower confidence levels (Error bars represent Standard Deviation).

At the behavioral level, participants (*N* = 17) reported they fully perceived the action (“4” in confidence level) in 40.2% of the trials (range: 10–72%), out of which 97.8% were indeed correctly reported (range: 92.3–100%). Participants reported they did not perceive the action at all (“1” in confidence level) in 36.1% of the trials (range: 14.6–83%), out of which 71.2% were indeed incorrectly reported (range: 62.71–86.75%). Intermediate levels of perception (“2” and “3” in confidence level), were reported in 14.4% and 9.4% of the trials, respectively (62.7% trials with confidence level “2” and 86.55% with confidence level “3” were correctly reported). These results imply that reports of confidence level correspond with performance accuracy (Fig. 2).

### Event‐related spectral perturbations

We first analyzed mu and beta rhythm power changes for trials in which participants correctly reported the actions with the highest confidence level (“Perceived” trials) and trials with incorrect responses with the lowest confidence level (“Nonperceived” trials).

**Figure 3:**
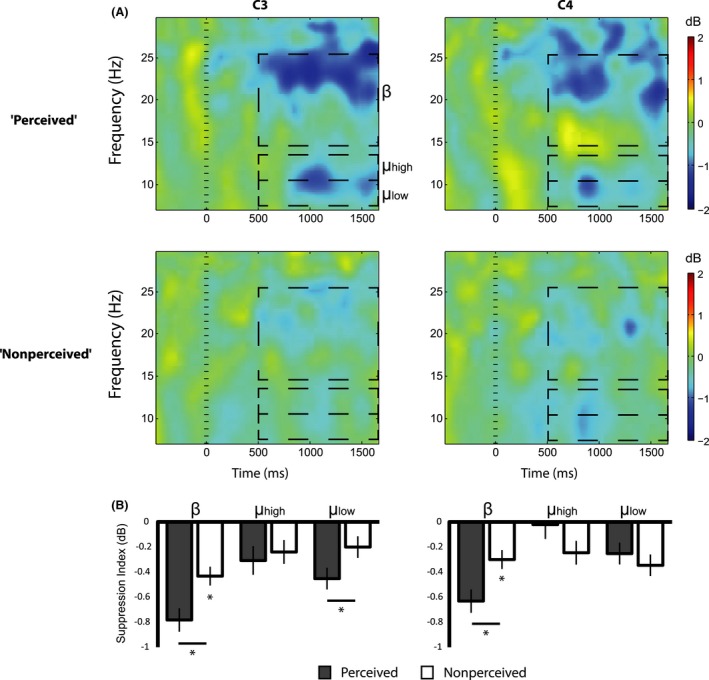
Mu and Beta suppression in Central sites (*N* = 17). Mu and Beta oscillation power for the “Perceived” and “Nonperceived” trials over the left (“C3”) and right (“C4”) sensorimotor cortices in the range of beta (15–25 Hz), high mu (11–13 Hz), and low mu (8–10 Hz). (A) Event‐Related Spectral Perturbations representing changes in oscillation power locked to target display (time 0 ms) relative to baseline (−500 to 0 ms). (B) Stronger suppression for perceptually perceived trials was found in bilateral beta and left low mu frequency ranges. Additionally, power in the beta range in both hemispheres was significantly suppressed in the Nonperceived trials relative to baseline (*P < 0.05 corrected, Error bars represent Standard Error).

**Figure 4:**
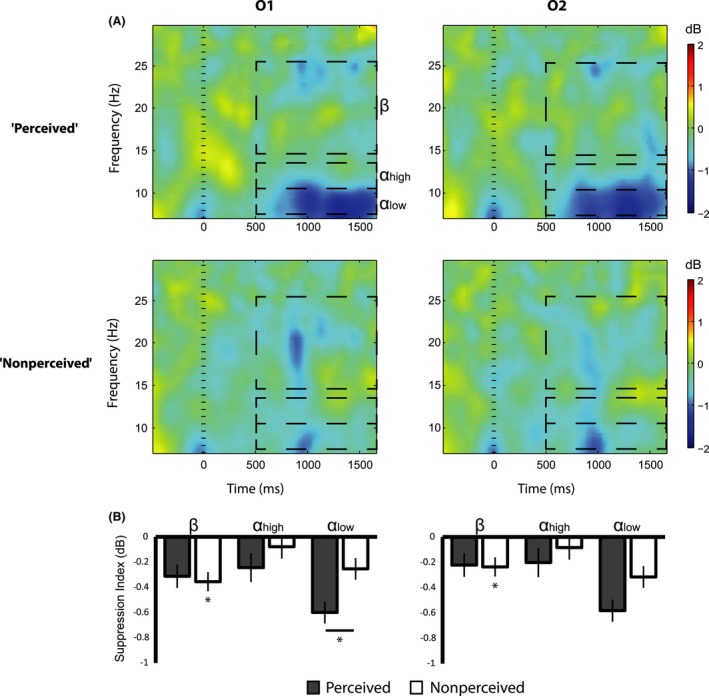
Alpha and Beta suppression in Occipital sites (*N* = 17). Alpha and Beta oscillation power for the “Perceived” and “Nonperceived” trials over the left (“O1”) and right (“O2”) visual cortices in the range of beta (15–25 Hz), high alpha (11–13 Hz), and low alpha (8–10 Hz). (A) Event‐Related Spectral Perturbations representing changes in oscillation power locked to target display (time 0 ms) relative to baseline (−500 to 0 ms). (B) Stronger suppression for the perceptually perceived trials was found only in the low alpha frequency range over O1 (and marginally significant in O2, see text). Power in the beta frequency was significantly suppressed in the nonperceived trials relative to baseline (*P < 0.05 corrected, Error bars represent Standard Error).

We analyzed the differences in suppression indices using repeated‐measures analysis of variance (ANOVA) with the factors of Condition (“Perceived”, “Nonperceived”), Channel (“C3”, “C4”, “O1”, “O2”), and Band (“Low Mu”, “High Mu”, and “Beta”). *P* values for effects that introduced violation of sphericity were corrected using lower‐bound epsilon values (Geisser and Greenhouse 1958). ANOVA showed significant main effects for all factors: Condition (*F*(1,16) = 10.03, *P* < 0.01), Channel (*F*(1,16) = 7.23, *P* < 0.01), and Band (*F*(1,16) = 5.00, *P* < 0.05). These main effects were qualified by third order interaction of Condition × Channel × Band (*F*(1,16) = 9.041, *P* < 0.01). This interaction was further examined with planned comparisons using one‐tailed pairwise *t*‐tests. Over both the left and right sensory motor cortices (channels C3 and C4), the oscillation power in the Beta range (15–25 Hz) was significantly lower in the Perceived versus Nonperceived trials (“Perceived” – “Nonperceived” Mean difference (Md) ± Standard Error of the Mean (SEM) = −0.35 ± 0.07 dB, *t*(16) = 2.47, *P* < 0.05 in C3 and −0.33 ± 0.02 dB, *t*(16) = 1.97, *P* < 0.05 in C4). Oscillation power in the low mu range (8–10 Hz) was significantly lower in the Perceived versus Nonperceived trials, but only over the left sensorimotor site (Md ± SEM = −0.25 ± 0.03 dB, *t*(16) = 1.92, *P* < 0.05 in C3 and 0.09 ± 0.03 dB, *t*(16) = 0.55, *P* = 0.31 in C4). In the higher mu frequency range (11–13 Hz), the oscillation power was not significantly different across trial types (Md ± SEM = −0.06 ± 0.02 dB, *t*(16) = −0.44, *P* = 0.33 in C3 and 0.22 ± 0.04 dB, *t*(16) = −1.34, *P* = 0.1 in C4) (Fig. 3). At the left and right occipital sites, oscillation power in the beta frequency range was not significantly different across perception levels (Md ± SEM = 0.04 ± 0.02 dB, *t*(16) = 0.35, *P* = 0.46 in O1 and 0.01 ± 0.02 dB, *t*(16) = 1.13, *P* = 0.44 in O2; one‐tailed, paired *t*‐test). The difference between perception levels in the low alpha range (8–10 Hz) was found significant in the left occipital site and marginally significant at the right occipital site (Md ± SEM = −0.34 ± 10^−3^ dB, *t*(16) = 1.86, *P* < 0.05 in O1 and −0.26 ± 0.07 dB, *t*(16) = 1.36, *P* = 0.09 in O2) (Fig. 4). In the higher alpha frequency range (11–13 Hz), the oscillation power was not significantly different across the two trial types (Fig. 4).

We further inspected each channel and band suppression indices in the “Nonperceived” trials relative to baseline using one‐tailed pairwise *t*‐tests (Bonferroni corrected for the four channels in each frequency band). Only suppression indices in the beta range were significant (M ± SEM = −0.42 ± 0.08 dB, *t*(16) = 5.05, *P* < 0.01 in C3, −0.32 ± 0.05 dB, *t*(16) = 3.11, *P* < 0.01 in C4, −0.35 ± 0.03 dB, *t*(16) = −2.03, *P* < 0.01 in O1, and −0.23 ± 0.04 dB, *t*(16) = −2.87, *P* < 0.01 in O2; one‐tailed paired *t*‐test) (Figs 3B and 4B).

**Figure 5:**
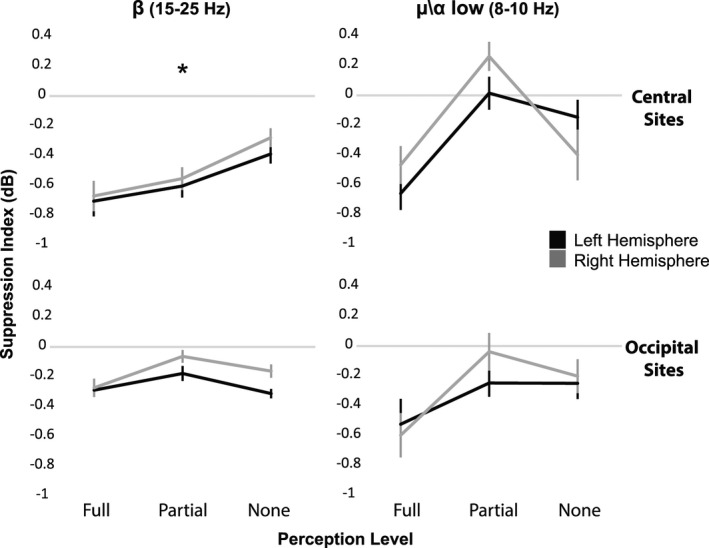
Power suppression in low Mu\Alpha and Beta (*N* = 11) and perception level. Low mu (8‐10 Hz) and beta (15‐25 Hz) oscillation power for the “Full”, “Partial”, and “None” perception level trials over the left (in dark) and right (in light) sensorimotor and occipital cortices. The graphs represent the changes in averaged suppression index across subjects at each of the three perception levels. A significant linear trend was found only for the averaged suppression index in the central sites at the beta range (*P < 0.05, Error bars represent Standard Error).

We also inspected to what degree the level of perception is correlated with the level of low mu and beta suppression. To that end, we computed the mu and beta suppression indices also for the partial perception trials ‐ all the trials in which the subjects reported partial (“2” or “3”) confidence level. Since partial levels of perception were reported in considerably fewer number of trials, only 11 subjects that passed the criterion of 25 trials per perception level (“full”, “partial”, and “none” perception levels) were included in this analysis. We observed a marginally significant within effect linear trend in the beta range over the left and the right sensory motor regions (*F*(1,10) = 3.98, *P* = 0.07 in C3 and *F*(1,10) = 3.55, *P* = 0.09 in C4) which was significant when each subject's suppression index was averaged across C3 and C4 (*F*(1, 10) = 5.42, *P* < 0.05). In the low mu range, we found a marginally significant linear trend but only over the left sensorimotor region (*F*(1,10) = 4.14, *P* = 0.08 in C3 and *F*(1,10) = 0.37, *P* = 0.84 in C4). Linear trends in the occipital sites were far from reaching significance (*F*(1,10) = 0.06, *P* = 0.8 in O1 and *F*(1,10) = 0.64, *P* = 0.43 in O2, in the beta range and *F*(1,10) = 1.10, *P* = 0.31 in O1 and *F*(1,10) = 1.62, *P* = 0.23 in O2 in the low mu range) (Fig. 5).

## Discussion

In this study, we investigated whether and to what degree activity in the mirror neuron system depends on conscious perception of actions. To this end, we rendered hand movement videos invisible to conscious perception and measured the magnitude of corresponding mirror activity via the modulation of EEG mu and beta oscillation power over the sensorimotor cortices. We found variations between the modulations of the two frequency bands. Oscillation power in the range of beta over both the right and left central sites, was significantly reduced during observation of conscious relative to unconscious perception of actions, as well as during unconscious action perception relative to baseline. Oscillation power in the range of mu, however, was significantly reduced only in the low range (8–10 Hz) and only in left central site, during conscious perception relative to unconscious perception. Relative to baseline, the mu oscillation power was not significantly suppressed.

Stronger mu and beta suppression over the bilateral sensorimotor cortices in conscious relative to unconscious perception have been reported in sensory modalities including the tactile and auditory domains. In a MEG study, mu and beta suppression over the bilateral primary somatosensory cortices were more suppressed between perceived versus not perceived tactile stimulation, and nonperceived tactile stimulation was significantly suppressed relative to baseline (Palva et al. 2005). Similarly, EEG oscillations in the mid‐frequency range (12–20 Hz), have been reported to reflect auditory illusion intensity, with stronger power reductions corresponding with stronger percepts (Leske et al. 2013).

In this study, we observed lateralized effects only in the low mu frequency band. Mu oscillation power was significantly more reduced for perceived versus not perceived actions only in the low range (8–10 Hz) and over the left central site. This lateralization is unlikely to reflect the fact that subjects used their right hand to report their subjective perception since subjects had to prepare equally to report perceived and nonperceived trials. Our result is in agreement with previous findings that addressed the mirror neuron system showing that observation of a moving hand elicits stronger mu suppression (Perry and Bentin 2009; Perry et al. 2011) as well as stronger fMRI signal response (Shmuelof and Zohary 2005; Lorey et al. 2013) in the hemisphere contralateral to the observed hand. Our stimuli consisted of right hand movements, compatible with a stronger effect over the left, contralateral hemisphere in the consciously perceived trials.

Our finding of significant suppression relative to baseline only in central beta during the “Nonperceived” trials, point to a functional dissociation between mu and beta with respect to conscious perception. Differences in oscillation power levels between these frequency bands during action observation were reported in other dimensions. For example, Avanzini and colleagues (Avanzini et al. 2012) demonstrated that only central beta oscillation power follows the temporal envelop of movement dynamics. This suggests the processing and representation of specific action kinematic aspects is carried by the beta rhythm. Other studies conducted by Hari and colleges with MEG add support to this assertion, demonstrating that the sources of beta and mu rhythms are different, as beta originate predominantly in the precentral primary motor cortex, whereas mu rhythms originate in the primary sensorimotor cortex (Hari et al. 1997; Hari 2006).

Our ability to claim that our results are specific to the mirror system activity is limited, as we did not examine suppression characteristics for nonaction visual stimuli. Nonetheless, we believe the current results are unlikely to be accounted for by general nonspecific response to visual stimuli. Studies that compared EEG responses to nonaction (e.g., words or symbols) perceived versus identical unperceived visual stimuli, report occipital to parietal power suppression in beta (Minami et al. 2014; Kloosterman et al. 2015) and alpha (Babiloni et al. 2006; Gaillard et al. 2009; Bazanova and Vernon 2014). Here we report functional differences between perceived and nonperceived action stimuli in central sites but not in occipital sites. Additionally, the differences and the linear trend we measured between perception levels at central sites in the beta range, were not observed at the occipital sites. Finally, the lateralization we measured between the left and right central sites in the low mu range, were not observed at occipital sites. Interestingly, an EEG study that examined the neural activity during conscious versus unconscious processing of tools, reported stronger mu (8–13 Hz) suppression over the left centro‐parietal regions for visible versus invisible tools conditions, and significant mu suppression for the invisible tool condition relative to baseline. Stronger suppression in beta was obtained for visible versus invisible tools as well (Suzuki et al. 2014). Passive observation of manipulable objects that afford possible actions, has been shown to elicit similar neural responses to passive observation of others' actions (i.e., Canonical Neurons, Creem‐Regehr and Lee 2005; Caggiano et al. 2009; Cisek and Kalaska 2010; Proverbio et al. 2011; Proverbio 2012). Taken together, the central mu and beta modulations we found for perceived versus nonperceived actions seem more likely to be specific to observed actions.

Our interpretation that mirror system activity depends on the level of action perception is compatible with EEG and MEG studies showing weaker suppression to visible but unattended actions. In these studies, subjects were presented with actions while they performed an orthogonal task. Attentional modulations were correlated with modulations both in mu (Perry and Bentin 2010; Schuch et al. 2010) and beta (Muthukumaraswamy and Singh 2008) oscillation power.

This study consisted of relatively limited number of trials as trial type depended on subjective perception, therefore single‐trial classification of perception level based on oscillatory power suppression was not feasible. Future studies using larger number of trials should address this issue and examine whether perception can be classified not only based on power suppression in response to the stimulus, but also predicted based on spontaneous prestimulus activity (i.e., oscillatory power prior to trial onset).

## Conclusion

Our data demonstrate that the degree of mirror neurons activity depends on the level of action perception, which is in line with theories associating the mirror system function with action understanding and understanding intentions of others. Yet, our data also imply that the mirror neuron system responds significantly to actions that are not consciously perceived, which indicates that such actions are also being processed to some degree. The behavioral effect of implicit imitation (e.g., the chameleon effect) is in line with such physiological responses.

## Conflict of Interest

None declared.
